# Epidemiologic features and burden of atopic dermatitis in adolescent and adult patients: A cross-sectional multicenter study

**DOI:** 10.1016/j.waojou.2021.100611

**Published:** 2021-12-06

**Authors:** Gloria Sanclemente, Natalia Hernandez, Daniela Chaparro, Liliana Tamayo, Angela Lopez, Colombian Atopic Dermatitis Research Group, Natalia Hernandez, Gloria Sanclemente, Daniela Chaparro, Ángela López, Andrés Cortes, Ángela Seidel, Clara Inés Ortiz, Claudia Arenas, Esperanza Meléndez, Julio Amador, Liliana Tamayo, Lina Colmenares, María Claudia Guzmán, María Claudia Torres, Mariela Tavera, Mauricio Torres, Miriam Vargas, Mónica Novoa, Mónica Rivera, Natalia Vélez, Oscar Mora, Oscar Medina, Paola Cárdenas

**Affiliations:** f Dermatosoluciones SAS, Colombia; g Group of Investigative Dermatology (GRID), University of Antioquia, Medellin, Colombia; h Pontificia Universidad Javeriana, Bogota, Colombia; i IPS Fototerapia Bojanini y López SAS, Colombia; j Centro Dermatológico Federico Lleras Acosta, Bogotá, Colombia; k Hospital Universidad del Norte, Barranquilla, Colombia; l Universidad Militar, Bogotá, Colombia; m Universidad Pontificia Bolivariana, Medellín, Colombia; n Universidad CES, Medellín, Colombia; o Fundación Ciencias de la Salud, Bogotá, Colombia; p Universidad del valle, Cali, Colombia; q Universidad CES, Medellín; a Group of Investigative Dermatology (GRID), University of Antioquia, Medellín, Colombia; b Dermatosoluciones SAS, Calle 90 # 19A 49 Cons 501, Bogotá, Colombia; c Pontificia Universidad Javeriana, Bogotá, Colombia; d Universidad Pontificia Bolivariana, Medellín, Colombia; e IPS Fototerapia Bojanini y López SAS, Bogotá, Colombia

**Keywords:** Atopic dermatitis, Burden, Epidemiology, Cross-sectional, Comorbidities, Direct costs

## Abstract

**Background:**

Atopic dermatitis (AD) is considered as one of the most frequent chronic skin conditions. Previous AD epidemiologic studies have been mainly retrospective and/or have been performed through surveys instead of in-person visits. Epidemiological studies concerning AD in Latin American countries are scarce.

**Objective:**

To describe sociodemographic and clinical features and the economic burden of AD on children and adult patients in Colombia through in-person visits.

**Methods:**

This was a cross-sectional study of 212 patients that included sociodemographic and clinimetric data. The diagnostic criteria of Hanifin and Rajka was used and data relating to disease distribution, disease severity (through the BSA: Body surface area; EASI: Eczema Area and Severity Index; SCORAD: Scoring Atopic Dermatitis), Fitzpatrick's skin phototypes, personal and familiar history of allergic diseases, previous treatments, and personal history of comorbidities, was collected.

**Results:**

Patient age range was 12–76, and 52.8% were female. Disease distribution was mainly flexural (19.6%). Early age start, Denni-Morgan fold, and infections tendency were more frequent in adolescents compared to adults. Mean age of diagnosis was 12 years old, AD diagnosis was made mostly by a dermatologist, 48.1% (102 patients) reported alcohol consumption, and 59% of consumers were heavy drinkers. Comorbidities found were: chronic rhinitis (68.9%) food allergy (32.5%), allergic conjunctivitis (29.7%), and asthma (28.8%). Around 81% earned less than $896 US dollars and 59% invested 6–30% of their monthly budget yearly, and 40% had work or school absenteeism. Mean scores of BSA, EASI, and SCORAD involvement were 32.6, 13.7, and 42.4, respectively.

**Conclusions:**

This study adds well-supported data through an in-depth clinical and economical characterization of Colombian adolescents and adult patients with atopic dermatitis and shows its high impact and burden on patients and their families. It also contributes to understand the burden of AD in Latin America.

## Introduction

Atopic dermatitis (AD) is an inflammatory and chronic skin disease, frequently associated with other atopic manifestations that can affect both children and adults. Worldwide prevalence of the disease calculated through the use of mailed questionnaires and not by in-person evaluations ranges from 0.2% in China at ages of 13–14 years through 24.6% in Colombia.^1^ It is considered one of the most frequent chronic skin conditions and several studies have documented a high burden of the disease, with an important impact on public health.^2^

The burden of AD has been well characterized, especially in the pediatric populations in developed countries;^3^^,^^4^ being associated with sleep disturbances, impact on social functioning, and choice of occupation, resulting in impaired quality of life (QoL).^5^ Similarly, among adult patients with AD, the burden includes not only its awkward skin symptoms,^6^ but also impaired mental health, and reductions in work productivity.^7^ In fact, this burden is higher in the severe presentation of the disease, which has been reported in 10%–34% of adult patients.^8^

It has been described that AD prevalence has been increasing over the last 4 decades in several developed European countries due to lifestyle and environmental factors,^9^ but it has also been increasing in developing countries of Asia, Africa, Latin America, and the Middle East;^10^ more likely because of increasing urbanization, pollution, western diet consumption, and obesity.^11^ Such rise by itself imposes a substantial psychosocial and economic impact on patients and relatives which also leads to the increase of the global burden from skin disease. Interestingly, more recent studies have shown a high exposure to allergens in tropical regions.^12^ In fact, a cross-sectional population-based study performed in 6 Colombian cities during 2009 and 2010 has shown a prevalence of atopic eczema of 14% (95% CI: 12.5–15.3%).^13^

Management of AD not only requires pharmacological treatments. It also involves the use of non-reimbursed skin care products and the daily use of emollients in addition to transport or accommodation expenditures, and not-entirely reimbursed medical and psychological consultations.^14^^,^^15^ Therefore, the aim of this study was to describe important sociodemographic and clinical features and the economic burden of the disease on patients and their families in children and adult patients in Colombia in order to contribute to Latin America well-supported AD data. Such information is important not only to perform an in-depth characterization of this population and its needs, but it is also crucial in order to design more effective management strategies among Latin America communities.

## Patients and methods

A cross-sectional, non-interventional observational study was performed, in which patients over 12 years of age with Colombian nationality, or foreigners residing for more than 15 years in the country, with a diagnosis of moderate to severe atopic dermatitis (defined by medical criteria), were included, with a convenience sampling approach. Patients with any mental disability were excluded. The recruitment period was 8 months (July 2019 to February 2020). Patients attended the dermatological consultation of private practice offices, health provider institutions, and hospitals from 6 different Colombian regions: North-East (Main city: Bucaramanga); North-West (Main city, coast: Barranquilla); Central area (Main cities: Bogota, Medellin, Armenia); South-West (Main city: Cali). Informed consent was obtained from all adult patients and a signed consent form was obtained from all children and their parents or guardians. This study was approved by the Ethics and Investigation Committee of the IPS Universitaria as stated in Act # 134 of May 29, 2019.

All the researchers were previously trained in the use of the questionnaire. Data collection was performed in a stepwise approach: Step 1. The collection of economic features regarding the cost of illness and demographic data such as age, marital status, gender, education level, living place, health insurance, socioeconomic classification, and social security type. Step 2. A complete physical examination was performed by a dermatologist from which the clinically defined findings and diagnoses were recorded. For AD diagnosis, we used the diagnostic criteria of Hanifin and Rajka.^16^ Data related to disease distribution, disease severity (through the BSA: Body surface area; EASI: Eczema Area and Severity Index; SCORAD: Scoring Atopic Dermatitis), Fitzpatrick's skin phototypes, personal and familiar history of allergic diseases, previous treatments, and personal history of comorbidities, were also gathered. Food allergy information was taken from patient's history but it had to be diagnosed by the treating physician. The percentage of BSA involved was categorized as clear (0%), mild (>0.1 to < 16%), moderate (16 to < 40%), and severe (40–100%), as previously described.^17^ For EASI scoring (range: 0–72), erythema, excoriation, swelling and lichenification on the face/neck, upper and lower extremities and trunk were evaluated. The EASI strata were classified as follows: 0 = clear; 0.1–1 = almost clear; 1.1–7 = mild; 7.1–21 = moderate; 21.1–50 = severe; 50.1–72 = very severe.^18^ For SCORAD scoring (range: 0–103), 8 body areas were evaluated for erythema, excoriation, swelling, oozing/crusting, lichenification, and dryness in addition to pruritus and sleeplessness and the extent of the disease was calculated using the “rule of nines”. The SCORAD strata were classified according to its scoring as follows: clear-mild 0–28.9, moderate 29–48.9, severe 49–103.^19^

The patients’ social security classification was defined according to the Colombian general social security health system, which operates in 2 main affiliation regimens: the contributory scheme (employees, independent workers) and the subsidized regime (lower income people). There is also a special regime which includes the military force, the police, all schoolteachers, and the Colombian national petroleum company (Ecopetrol).

Socioeconomic classification was performed through a surrogated marker for individual or family income according to the National Administrative Department of Statistics (DANE),^20^ which has been based on public services payment related to housing type and urban or rural environment. Such classification has been stratified as: Stratum 1: Low-Low; Stratum 2: Low; Stratum 3: Medium-Low; Stratum 4: Medium; Stratum 5: Medium-High; Stratum 6: High.^20^

The cost of illness was determined according to the value of the resources expended by the patients as a result of their health disease. The exploration of economic expenses was done with both parents (when applicable) as in Colombia it is not unusual to find that the mother is the sole head of the household.

Income and illness investment were asked on a monthly base, and the costs related to treatment or in clothes, syndets, gloves, etc, were based on last year expenditures. All these data were included in the analysis.

### Statistical analysis

A univariate analysis was performed to describe variables‘ frequency. For continuous quantitative variables that fulfilled the assumption of normality (Kolmogorov Smirnov), the mean was used as a measure of central tendency and the standard deviation as a measure of dispersion. For those quantitative variables whose distribution was not normal, the median and the minimum and maximum values were used for the presentation of the data. Absolute and relative frequencies and percentages were used for qualitative variables. A Spearman correlation test and a Chi-square test of independence were performed to compare BSA with EASI, and SCORAD and to compare EASI with SCORAD scores and severity strata, respectively. Bivariate analysis was performed by the Student's t-test, one-way ANOVA, Chi-square, or U of Mann Whitney, as appropriate. The significance level was specified at 0.05 for all tests. For data processing, the SPSS (Statistical Package for Social Sciences) version 27, was used.

## Results

A total of 212 patients were included in this study. The mean age of participants was 28.5 years-old (range: 12–76 years). The main sociodemographic characteristics are presented in Table 1, where it should be noted that the population was made up mainly by females (52.8%), between 12 and 76 years old, with skin phototypes from II to IV (96.7%). The predominant marital status was being single (70.3%), and 68.9% of patients reported having a technical or professional degree. With regard to health insurance, the majority of patients (84%) belonged to the contributory regime. Regarding the economic characterization, 29.9% of the patients belonged to the medium-low stratum, 22% to the low stratum, 20.1% to the medium stratum, 15.9% to the medium-high stratum, 11.2% to the highest stratum and 0.9 to the low-low stratum. Gender was not associated with disease severity categories (measured either with EASI or SCORAD) (p = 0.09 and p = 0.43, respectively; Chi^2^ Test of Independence).

**Table 1:** Sociodemographic characteristics of Colombian atopic dermatitis patients

Variable	n = 212
Sex female, *n (%)*	112 (52.8)
Age, years, *mean (SD)*	
12–18 years	53 (25)
>18 years	159 (75)
Skin Phototype, n (%)	
II	32 (15.1)
III	116 (54.7)
IV	57 (26.9)
V	7 (3.3)
Marital status, *n (%)*	
Single	149 (70.3)
Married	45 (21.2)
Living common-law	13 (6.1)
Divorced	5 (2.4)
Highest educational level completed^a^, *n (%)*	
Elementary School	7 (3.3)
Middle/High School	59 (27.8)
Technician	29 (13.7)
College	78 (36.8)
Postgraduate	39 (18.4)
Social security classification^b^, *n (%)*	
Contributory	178 (84)
Subsidized	32 (15.1)
Special regimen	9 (4.2)

a Referred by the patient.

b Colombian general social security health system.

Regarding the clinical aspects and criteria for diagnosis shown in Table 2, disease distribution was mainly flexural (19.6%), combined with either eyelid dermatitis (11.7%), hand eczema (5.1%) or cheilitis (5.1%). In addition, early age start, Denni-Morgan fold and infections tendency were all more frequent in adolescents compared to adults. (p = 0.010 and p = 0.013, respectively; Chi-Square test).

**Table 2:** Clinical criteria for the diagnosis of atopic dermatitis in Colombian adolescent and adult atopic dermatitis patients

Variable, n (%)	n = 212	<18 years old n = 47	>18 years old n = 165
Xerosis	208 (98.11)	46 (97.87)	162 (98.18)
Itching when sweating	160 (75.47)	36 (76.60)	124 (75.15)
Course influenced by environment and emotions	151 (71.23)	28 (59.57)	123 (74.55)
Early age start	147 (69.34)	40 (85.11)	107 (64.85)
Intolerant to wool and organic solvents	105 (49.53)	22 (46.81)	83 (50.30)
Periorbital darkening	104 (49.06)	26 (55.32)	78 (47.27)
Denni – Morgan infraorbital folds	100 (47.17)	30 (63.83)	70 (42.42)
Elevated IgE	93 (43.87)	15 (31.91)	78 (47.27)
Anterior neck folds	87 (41.03)	22 (46.81)	65 (39.39)
Ichthyosis/palmar hyperlinearity	86 (40.57)	16 (34.04)	70 (42.42)
Cutaneous infections tendency	81 (38.21)	25 (53.19)	55 (33.33)
Cheilitis	77 (36.32)	14 (29.79)	62 (37.58)
Cutaneous tests reactivity	75 (35.87)	13 (27.66)	62 (37.58)
Facial erythema or paleness	71 (33.49)	21 (44.68)	55 (33.33)
Recurrent conjunctivitis	69 (32.55)	11 (23.40)	58 (35.15)
Food intolerance	67 (31.60)	10 (21.28)	57 (34.55)
Hand and foot dermatitis	58 (27.36)	10 (21.28)	48 (29.09)
Pityriasis alba	51 (24.06)	12 (25.53)	39 (23.64)
Nipple eczema	50 (23.58)	6 (12.77)	44 (26.67)
Follicular accentuation	49 (23.11)	12 (25.53)	37 (22.42)
White Dermographism	20 (9.43)	4 (8.51)	16 (9.70)
Keratoconus	10 (4.72)	1 (2.13)	9 (5.45)
Subcapsular anterior cataract	4 (1.89)	0 (0.0)	4 (2.42)

Mean scores of BSA, EASI, and SCORAD involvement were 32.6, 13.7, and 42.4, respectively. Body-surface area, EASI, and SCORAD absolute scores were correlated with each other (All comparisons: Rho>0.7; p = 0.00, Spearman correlation test) when BSA was compared with EASI, with SCORAD and when EASI was compared with SCORAD.

Mild, moderate and severe BSA scores were found in 29.7%, 35.4%, and 32.55% of patients, respectively. According to EASI scores, 33%, 39.6%, and 21.7% of the patients had mild, moderate or severe disease. Similarly, clear-mild, moderate and severe disease according to SCORAD were found in 24.5%, 36.8%, and 38.7% of the population.

When BSA strata were compared to EASI and to SCORAD, there was a statistical significant dependence of severity levels (p = 0.00 and p = 0.00, respectively; Chi^2^ Test).

With regard to the clinical and lifestyle aspects shown in Table 3, the median age of diagnosis of AD was 10 years old, being the diagnosis of AD made by a dermatologist in more than half of the cases (55.7%). About half of the patients reported alcohol consumption (48.1%), while smoking was found in less than 1 in every 10 patients (7.5%). The most common comorbidities were chronic rhinitis (68.9%), sleep disturbances (48.6%), asthma (39.6%), anxiety (20.7%), and depression (16.5%).

**Table 3:** Clinical aspects of Colombian adolescent and adult atopic dermatitis patients

Variable	n = 212	<18 years old n = 47	>18 years old n = 165
Age at diagnosis, *median*	10	15	29
Specialist or physician who had made the diagnosis, *n (%)*			
Dermatologist	118 (55.7)	21 (44.7)	97 (58.8)
General physician	37 (17.5)	12 (25.5)	25 (15.1)
Pediatrician	33 (15.6)	8 (17.0)	25 (15.1)
Allergologist	17 (8)	6 (12.8)	11 (6.7)
Other	7 (3.3)	0 (0.0)	7 (4.2)
Habits, *n (%)*			
Alcohol	102 (48.1)	3 (6.4)	99 (60)
Smoker	16 (7.5)	0 (0)	16 (9.7)
Psychoactive substances	6 (2.8)	0 (0)	6 (3.6)
History of psychiatric symptoms^a^, *n (%)*			
Anxiety	44 (20.7)	12 (25.5)	32 (19.4)
Depression	35 (16.5)	7 (14.9)	28 (16.7)
Attention deficit	19 (9.0)	4 (8.5)	15 (9.1)
Sleep disturbances^a^, *n (%)*	103 (48.6)	21 (44.7)	82 (49.7)
Disease severity, *mean (SD*^b^*)*			
SCORAD	42.4 (19.4)	45.36 (20.3)	41.57 (19.2)
EASI	13.7 (11.8)	14.26 (12.5)	13.57 (11.6)
BSA	32.5 (25.0)	33.33 (24.8)	32.42 (25.2)

a Referred by the patient.

b SD: standard deviation; SCORAD: scoring atopic dermatitis; EASI: eczema area and severity index; BSA: body surface area.

Comorbidities, previous treatments and economic features are depicted in Table 4, Table 5, Table 6, respectively. Economic dependence, socioeconomic strata and monthly income were all associated with age strata (p = 0.00, 0.00, 0.001, respectively; Chi^2^ Test), but no significant dependence was found with monthly investment in the disease (p = 0.08; Chi^2^ Test). In addition, neither socioeconomic strata, economic dependence monthly income nor monthly disease investment were associated with disease severity (measured either with EASI or SCORAD) (All results p > 0.005; Chi^2^ Test).

**Table 4:** Comorbidities in Colombian adolescent and adult atopic dermatitis patients

Variable	n = 212	<18 years old n = 47	>18 years old n = 165
Atopic Comorbidities, *n (%)*			
Allergic rhinitis	146 (68.9)	37 (90.2)	109
Asthma	61 (28.8)	12 (25.5)	49 (29.7)
Food allergy	69 (32.5)	12 (25.5)	57 (34.5)
Allergic conjunctivitis	63 (29.7)	11 (23.4)	52 (31.5)
Metabolic Comorbidities, *n (%)*			
Thyroid disease	8 (3.8)	0 (0.0)	8 (5)
Arterial hypertension	8 (3.8)	0 (0.0)	8 (5)
Obesity	5 (2.4)	0 (0.0)	5 (3)
Diabetes	4 (1.9)	0 (0.0)	4 (2.4)
Dyslipidemia	1 (0.5)	0 (0.0)	1 (0.6)
Other Comorbidities, *n (%)*			
Peptic ulcer	5 (2.4)	1 (2.1)	4 (2.4)
COPD^a^	1 (0.5)	0 (0.0)	1 (0.6)
OSA	1 (0.5)	0 (0.0)	1 (0.6)

a COPD: chronic obstructive pulmonary disease; OSA: Obstructive sleep apnea.

**Table 5:** Treatment received in Colombian adolescent and adult atopic dermatitis patients

Variable	n = 212	<18 years old n = 47	>18 years old n = 165
Topical treatments, *n (%)*			
Moisturizers	209 (98.6)	47 (100)	162 (98.2)
Topical corticosteroids	206 (97.2)	46 (97.9)	160 (96.7)
Topical tacrolimus	126 (59.4)	20 (42.5)	106 (64.2)
Systemic treatments, *n (%)*			
Oral antihistamines	183 (86.3)	41 (87.2)	142 (86)
Systemic steroids	133 (62.7)	23 (48.9)	110 (66.6)
Phototherapy	131 (61.8)	28 (59.5)	103 (62.4)
Azathioprine	60 (28.3)	7 (14.9)	53 (32.1)
Cyclosporine	47 (22.2)	6 (12.7)	41 (25)
Methotrexate	36 (17.0)	8 (17)	28 (17)
Mycophenolate mofetil	8 (3.8)	2 (4.2)	6 (3.6)
Biologic therapy, *n (%)*			
Dupilumab	14 (6.6)	0 (0.0)	14 (8.5)
Omalizumab	12 (5.7)	3 (6.3)	9 (5.5)

**Table 6:** Economic aspects in atopic dermatitis patients in Colombia by age

Variable, n (%)	Age			
	Total n = 212	12-<18 years n = 53	18–65 years n = 155	>65 years n = 4
**Economic dependence**				
Yes	128 (59.8)	55 (100)	72 (46.6)	1 (25)
No	86 (40.2)	0 (0)	83 (53.5)	3 (75)
**Monthly income, CCMMW**^a^				
No income	91	44	47	0
<1	15	1	14	0
1–2	34	5	28	1
3–4	34	3	31	0
5–6	16	0	16	0
7–10	10	0	8	2
10–15	8	1	7	0
16–20	4	1	3	0
>21	2	0	1	1
**Monthly investment in disease, USD**^b^				
0–25	31	1	29	1
25–50	57	16	40	1
50–130	95	31	63	1
130–260	22	4	17	1
>260	2	2	0	0
Patient does not know	7	1	6	0

a CCMMW: Current Colombian monthly minimum wage. One CCMMW is equivalent to approximately 224 US dollars.

b USD: US dollars.

In addition, another relevant finding was that 37% of adult patients sought alternative medicine as other therapy option.

Finally, almost 40% of the patients had work or school absenteeism, mostly for 1–4 days (46 patients out of 212) followed by 5–8 days (15 patients out of 212) and less frequently for more than 9 days in the last 6 months.

## Discussion

The current study demonstrated major economic and health status impairments in a representative population of adolescent and adult patients with atopic dermatitis in Colombia. We characterized these patients with regards to socio-demographic variables, habits, and comorbidities, with alcohol consumption, and sleep disturbances (48.6%), as main concerns in this population. One of the first reports of AD in Colombia that is now more than 10 years-old focused on asthma and other allergic conditions in which AD symptoms were assessed through a well-performed school-based study with a multistage cluster sampling from Colombian private and public schools.^13^ Another Latin America study included at least 1000 children that were selected predominantly by a random sample of schools within the study area but such study also used self-respondent questionnaires of the International Study of Asthma and Allergies in Childhood (ISAAC) that assessed the symptoms of atopic eczema.^21^ A more recent study performed in an allergy referral center in Medellin, Colombia has described several risk factors for severe and persistent dermatitis and allergen sensitization in 94% of evaluated patients.^22^ Our study in comparison included adolescents (25%) and adult patients (75%) from 6 Colombian regions who were assessed in-person by experienced dermatologists what assured an objective characterization of the severity of the disease through the use of previously validated scales such as BSA, SCORAD, EASI, and at the same time, and a differential factor from other studies, it included an in-depth assessment of important sociodemographic characteristics as well as previous treatments and the cost of illness for patients and their families.

The mean age of interviewed patients was 28.5 years, a finding that is very relevant in this study, as this is the age at which an individual's productivity and labor could be more affected by the disease. In addition, the mean age of diagnosis of AD of our patients was at 10 years-old, a finding that contrasts with a previous report of patients from Medellin.^22^ In this respect, since the age of AD diagnosis was gathered through patient history, recall bias may have occurred, although we think it might be unlikely as the disease is such a stressful condition that most patients or their parents will probably remember when their life (or children's life) changed with the onset of atopic dermatitis.

With regard to gender, we found a slight female preponderance, which has been also reported in other studies.^1^^,^^23^^,^^24^ This finding could be explained due to the predominance of adult patients, as several studies have found that AD is more frequent in boys during childhood, but it starts to be more frequent in females during adolescence and thereafter.^24^^,^^25^ However, it remains unclear why AD seems to be more frequent in women compared to men. Another interesting result regarding gender was its lack of association with AD disease severity, a finding that is similar to what has been reported in children and in adults with AD^19^^,^^26^ although a higher prevalence of eczema has been found in women when compared with men.^24^^,^^27^

A main concern in our study is the preponderance of being single and the important percentage of patients with anxiety and depression, findings that could suggest a negative impact of AD in formal relationships probably due to psychosocial stressors associated with the disease. Such impact has also been described in other populations in which never-married adults and higher rates of divorce and separation have been found.^28^^,^^29^

Regarding the level of education, most of the included individuals finished school, high school, or college or had a technician or university degree. Although the level of education of the Colombian population tends to be lower, this finding could be explained by the inclusion of patients mostly with the contributive social security which in our country usually corresponds to people with a formal job, representing a better income.

Initial diagnosis of AD varies according to each country. Our study shows that more than half of the patients were diagnosed by a dermatologist (55.7%) followed by general practitioners (GP) (17.5%) and less frequent by pediatricians and allergologists with no differences between adolescents and adult patients. These findings contrast with other countries such as France, Italy, Germany, Spain, and the United Kingdom where patients are usually first diagnosed by their GPs.^30^ Although we do not have an explanation for such results we assume it has to do with the economic effort they make to consult a dermatologist in private practice or either with the level of dermatology training that other physicians receive during their medical career which is scarce in most medicine curricula in Colombia or with the lack of trained specialists in the management of AD as has been reported in developing countries.^10^

Sleep deprivation, itch, and skin lesions localized mainly on visible areas can all cause social embarrassment and psychosocial alterations. We have confirmed that AD not only can have a detrimental effect on sleep, but it is also related with heavy alcohol intake, a finding that deserves further attention not only because it was more frequent than depression and anxiety (the 2 main psychiatric conditions usually reported), but also because it has been more frequently described only in recent years in patients with eczema and other inflammatory diseases such as psoriasis.^31^

In addition, our study also supports the already known link between AD and other allergic conditions such as asthma, rhinitis, and mite allergy^22^^,^^32^ in both, adolescents and adults. This is also consistent with several reports of the “allergic march”, in which AD has been shown to precede the appearance of other atopic conditions such as asthma, chronic rhinitis, and food allergy.^33^^,^^34^

Except for early age onset, recurrent infections and the Denni-Morgan infraorbital folds, there were no important differences in the majority of clinical variables evaluated in adolescent and adult patients, a result that contrasts with previous reports in which significant differences in atopic history, signs, and symptoms of <18 years in comparison to adult patients have been found.^35^^,^^36^ Such findings could be explained by the age-proximity of the majority of studied adolescents to adulthood which could in turn lead to phenotypical similarities.

An accurate assessment of the severity and extent of AD is crucial not only to define its burden but also to evaluate the effects of treatment. In this study, we have assessed the severity of the disease through three of the main available instruments: BSA, EASI, and SCORAD. Interestingly, we have found a significant correlation between all scales when using absolute values and also when stratified scales were compared, findings that contrast with what has been reported previously.^37^ In addition, it is important to highlight that although at the time of evaluating the patients, the severity scales yielded scores corresponding to mild to moderate disease, it must be taken into account that almost all the patients were receiving either systemic, phototherapy, or biological treatment (Table 5), which influenced the finding of lower than expected scores when severity scales were applied. In this latter respect, we also confirm that the majority of adolescent and adult patients required the continuous use of emollients, topical corticosteroids, anti-histamines, and/or topical calcineurin inhibitors and that more than half of our adult patients have required systemic corticosteroids and other immunosuppressants such as cyclosporine or methotrexate, all of which put our patients at risk due to their already known side effects, particularly infection. These findings of the severity of the disease warrants strict monitoring of these patients as an elevated mortality has been reported among patients with the most severe or active types of eczema.^38^

As AD is well known for its chronicity and frequent exacerbations, not surprisingly, our study showed an important use of alternative medicine, a result that confirms the frustration and dissatisfaction of our patients with conventional treatment. All these contrast with the low use of a US Food and Drug Administration (FDA) approved biologic for AD such as dupilumab, a fact that can be explained by its recent launching into the Colombian market.

With respect to income, the majority of our patients belonged to lower income strata (Stratum 4 or less), a finding that has been also described in the adult population of the United States.^27^^,^^39^ This finding is very important, given that in a previous Colombian multicenter study, a higher impact on QoL was found in the lower income strata among patients with different skin diseases.^40^ Other AD costs include physician visits, prescriptions, and hospital, emergency or non-prescription expenditures whereas indirect costs include impaired work productivity (presenteeism) and absenteeism from work.^41^ This was confirmed in our study in which not only 40.2% of patients have had work or school absenteeism, but also almost 60% of our population was economically dependent. In addition, 81% earned less than $896 US dollars (4 Current Colombian monthly minimum wage) and 59% invested around 6–30% of their monthly budget in their AD (US$300–3120) yearly, a result that exceeds the out-of-pocket expenses reported in a US community survey^42^ and also the costs on health-care of atopic eczema from a recent survey performed in all Europe.^30^ These findings are also reinforced by the fact that disease monthly investment was not different in adolescents vs adults. All these can be explained not only by our health system structure in which moisturizers and syndets are scarcely provided to patients and by the high cost of those products and of hypoallergenic or non-scented personal hygiene products. Also, 40% of the patients had work or school absenteeism during the last 6 months, most of them for 1–4 days, which is in line with the world literature regarding the indirect costs of the disease.^43^

Not surprisingly, there were significant differences in economic dependence, socioeconomic strata, and monthly income according to age since it is expected that as people advance in age, their income and socioeconomic stratum improve and they become less economically dependent. In addition, none of these economic variables were influenced by the severity of AD which is in line with what has been reported in Europe, as patients suffering milder disease also incur very high extra costs to cover emollients, moisturizers, and not-reimbursed treatments.^15^

The strength of this study relies not only on the in-person clinimetric evaluation of patients by experienced dermatologists but also on its target on patients recruited from different sources at the main cities in Colombia, and on its in-depth clinical and economical characterization of the disease, as previous worldwide AD data have been mainly collected through self-reported questionnaires sent by mail.^1^

The limitation of our study was its probable selection bias due to the cross-sectional nature of the study and because patients with a history of moderate-severe disease may have participated at higher rates than those with mild AD. Additionally, and in relation to the aforementioned, we cannot know the reasons why other AD patients did not seek health care at the time we were developing the study.

## Conclusion

This study highlights the significant disease burden associated with atopic dermatitis in a Latin American adolescent and adult population, suggesting the need for future research focused on AD effects on QoL, its risk factors, and therapeutic and prevention strategies.

## Abbreviations

**AD**, Atopic dermatitis; **BSA**, Body surface area; **EASI**, Eczema Area and Severity Index; **SCORAD**, Scoring Atopic Dermatitis; **DANE**, National Administrative Department of Statistics (Colombia); **ISAAC**, International Study of Asthma and Allergies in Childhood; **GP**, General practitioners; **FDA**, Food and drugs administration; **US**, United States.

## Ethical statement

All authors assure that in this study the following has been fulfilled:

1) This material is the authors' own original work, which has not been previously published elsewhere.
2) The paper is not currently being considered for publication elsewhere.
3) The paper reflects the authors' own research and analysis in a truthful and complete manner.
4) The paper properly credits the meaningful contributions of co-authors and co-researchers.
5) The results are appropriately placed in the context of prior and existing research.
6) All sources used are properly disclosed and cited.
7) All authors have been personally and actively involved in substantial work leading to the paper, and will take public responsibility for its content.

The project in which this study is framed (Title: “CARGA DE LA ENFERMEDAD Y CALIDAD DE VIDA DE PACIENTES ADULTOS Y ADOLESCENTES CON ECCEMA ATÓPICO: CARACTERIZACIÓN CLÍNICA Y SOCIODEMOGRÁFICA”) and its informed consent were both approved by the Ethics and Investigation Committee of the IPS Universitaria as stated in Act # 134 of May 29, 2019.

## Authors contributions

**Gloria Sanclemente**: Conception and design of the study, methodology**,** acquisition of data, original and draft preparation, analysis and interpretation of data, writing-reviewing and editing of the manuscript, final approval of the submitted version. **Natalia Hernandez:** Conceptualization, acquisition of data, writing-original draft preparation. **Daniela Chaparro:** Conceptualization, statistical analysis, writing-original draft preparation, final approval of the submitted version. **Liliana Tamayo:** Acquisition of data, writing-original draft preparation, final approval of the submitted version. **Angela Lopez:** Acquisition of data, writing-original draft preparation, final approval of the submitted version.

## Funding

This study was sponsored by the Asociación Colombiana de Dermatología y Cirugía Dermatológica (Asocolderma).

## Authors’ consent for publication

All authors have consented to publication of the study.

## Availability of data and materials

The data that support the findings of this study are available by request from the corresponding author. The data are not publicly available due to ethics committee/informed consent restrictions regarding the use of data from a third party, and due to participants’ privacy issues.

## Declaration of competing interest

This study was sponsored by the Asociación Colombiana de Dermatología y Cirugía Dermatológica (Asocolderma) through a Sanofi Laboratories grant. However, this pharmaceutical laboratory has not participated in any workshop related to the study and has not being involved in the analysis or design and development of the study.
